# Trogocytosis between Non-Immune Cells for Cell Clearance, and among Immune-Related Cells for Modulating Immune Responses and Autoimmunity

**DOI:** 10.3390/ijms22052236

**Published:** 2021-02-24

**Authors:** Ko-Jen Li, Cheng-Han Wu, Cheng-Hsun Lu, Chieh-Yu Shen, Yu-Min Kuo, Chang-Youh Tsai, Song-Chou Hsieh, Chia-Li Yu

**Affiliations:** 1 Department of Internal Medicine, National Taiwan University Hospital and National Taiwan University College of Medicine, Taipei 10002, Taiwan; dtmed170@yahoo.com.tw (K.-J.L.); chenghanwu@ntu.edu.tw (C.-H.W.); b89401085@ntu.edu.tw (C.-H.L.); tsichhl@gmail.com (C.-Y.S.); 543goole@gmail.com (Y.-M.K.); 2 Institute of Clinical Medicine, National Taiwan University College of Medicine, Taipei 10002, Taiwan; 3 Division of Allergy, Immunology & Rheumatology, Taipei Veterans General Hospital, Taipei 11210, Taiwan; cytsai@vghtpe.gov.tw

**Keywords:** trogocytosis, oncologic trogocytosis, amoebic trogocytosis, cell clearance, tumor surveillance, tumor evasion, chimeric antigen receptor T lymphocyte, antibody-dependent cell-mediated cytotoxicity, antigen modification, immune plasticity

## Abstract

The term trogocytosis refers to a rapid bidirectional and active transfer of surface membrane fragment and associated proteins between cells. The trogocytosis requires cell-cell contact, and exhibits fast kinetics and the limited lifetime of the transferred molecules on the surface of the acceptor cells. The biological actions of trogocytosis include information exchange, cell clearance of unwanted tissues in embryonic development, immunoregulation, cancer surveillance/evasion, allogeneic cell survival and infectious pathogen killing or intercellular transmission. In the present review, we will extensively review all these aspects. In addition to its biological significance, aberrant trogocytosis in the immune system leading to autoimmunity and immune-mediated inflammatory diseases will also be discussed. Finally, the prospective investigations for further understanding the molecular basis of trogocytosis and its clinical applications will also be proposed.

## 1. Introduction

Trogocytosis (from the ancient Greek word “trogo”, meaning gnaw) involves the transfer of plasma membrane fragments between two cells in contact. It is a means for intercellular communication. Many investigators believe that trogocytosis first appeared in very primitive organisms as a way to directly transfer some regulatory molecules thus contributing to the establishment of intercellular communications [1]. Trogocytosis is proved an active and rapid transfer process after conjugation formation between two homogeneous or heterogeneous living cells. Therefore, it is different from phagocytosis of apoptotic bodies. After trogocytosis, the two living cells may detach with the obtained membrane fragments for continuous communication. Partial phagocytosis, cell nibbling or cannibalization is also different names for trogocytosis [2]. This cellular growth behavior was first observed among amoebas [3,4] and then between cells of the immune system [5].

The biological significance of trogocytosis includes: (1) cell-cell information exchange, (2) growth during embryonic development, (3) immunoregulation, (4) nibbling to death of infectious agents, (5) trogo-transit of intracellular parasites, and (6) cancer immunology [6]. Obviously, the immune system is tremendously affected by trogocytosis to exhibit its diverse effector functions. The immune system cells can be arbitrarily divided into innate (monocytes, macrophages, and granulocytes) and adaptive immune cells (T lymphocytes, B lymphocytes and natural killer cells, NK). The interactions between innate and adaptive immune cells rely on cell-cell contact with trogocytosis and a network of released cytokines/chemokines/growth factors. The innate immune cells are not merely phagocytic cells but act as antigen-presenting cells (APCs) by presenting endogenous and exogenous antigens to adaptive immune cells for eliciting cell-mediated (T cells) and antibody-mediated (B cells) immune responses depending on the antigen nature. T lymphocytes are further classified into several subsets including CD4^+^T (helper T cells), CD8^+^T (cytotoxic T cells), CD17^+^T (inflammatory and autoimmune T) and regulatory T (Treg). NK are non-T, non-B cells that act in tumor cell killing without previous sensitization and for immunosuppression. Granulocytes contain polymorphonuclear neutrophils (PMN) that act as professional phagocytes as well as cytokine-producing cells. Basophils can induce allergic reactions by releasing vasoactive amines. Eosinophils are involved in allergy and parasitic infestations. In this review, we will discuss in detail these six aspects. We will also discuss the involvement of abnormal trogocytosis in different autoimmune, rheumatic inflammatory and metabolic diseases. Finally, we will discuss some current enigmas in trogocytosis and propose some future clinical applications.

## 2. Trogocytosis between Embryonic Cells for Un-Wanted Cell Clearance, Germ Cell Remodeling and Sperm Elimination in Embryonic Development

It is widely recognized that multicellular organisms routinely remove unwanted, excessive or aging cells from the body. This important process is called cell clearance. Cell clearance appears to be evolutionarily conserved [7,8]. Traditionally, cell clearance is mediated by phagocytosis of apoptotic cells, either by professional phagocytes or non-professional phagocytes, such as epithelial cells and fibroblasts [9]. Recently, a mechanism of partial cell eating or cannibalism, was proposed by Abdu et al. [2]. These authors documented that the developing *C. elegans* embryo endodermal cells could actively ingest lobes from primordial germ cell (PGC) bodies, but did not scavenge the PGC lobe debris. Most importantly, this cannibalism leaves the rest of PGC viable but affects the size and cell composition of PGCs during development. The authors also observed that CED-10/Rac-1-induced actin, DYN-Y dynamin, and LST-4/SNX9 may transiently surround the lobe necks and were required by endothelial cells for lobe scission. This evolutional observation defines a new form of developmental programmed cell remodeling involved in the intercellular cannibalism to shape cells via embryonic trogocytosis [10]. Furthermore, Weinhard et al. [11] defined a set of dynamic microglia-synapse interactions including the selective partial phagocytosis, or trogocytosis of presynaptic structure and the induction of postsynaptic spine head filopodia by microglia in developing organotropic hypocampal cultures. The results may suggest that microglia cells are highly motile cells proposed for synaptic nibbling during neuronal circuit formation. Recently, Villano et al. [12] proposed the current view that microglia can nibble the entire synapses and highlighted the complexity of neuronal-microglial interactions in vivo.

Although the cell-cell contact with nibbling or cannibalism is characteristic for trogocytosis [13,14], the behavior of separating the adhesive receptor-ligand complex between the two opposing cells is a unique property of this biological process [15,16]. Ralston et al. [17] reported that ephrin receptor (Eph) tyrosine kinases and their membrane-bound ephrin ligands are the prominent inducers of contact-repulsion during embryonic development resembling embryonic trogocytosis. Gong et al. [18] showed that the phagocytic adaptor protein Gulp 1 can regulate EphB/ephrin B trogocytosis for activating efficient cell rearrangements of the cultured cells during embryonic development. Gulp 1 could mediate trogocytosis bidirectionally by dynamic engagement with EphB/ephrin B protein clusters in cooperation with Rac-specific guanine nucleotide exchange factor Tiam 2. The authors concluded that Gulp 1 presence at the Eph/ephrin cluster was a prerequisite for recruiting the endocytic GTPase dynamin. Trogocytosis is considered a unique phagocytosis-like phenomenon to perform effective membrane scission and engulfment. Besides, PMN was proved to kill the unicellular flagellated parasites by taking cell membrane fragments mimicking cell cannibalism [19,20]. In addition, Olivera-Valle et al. [21] revealed that vaginal PMNs bit sperms and quickly reduced sperm motility (<5 min) and viability (<20 min) after cell-cell contact in the vaginal lumen with a low impact on the mucosa.

## 3. Trogocytosis-Associated Cytopathic Effects, Immune Evasion and Immune Response Depending on Different Pathogenic Microbes

### 3.1. Cytopathic Effects by Eukaryotic Amoebic Parasites via Trogocytosis

Trogocytosis was firstly described in eukaryotic microbe amoebae in killing host eukaryotic cells. Brown [22] observed that the “brain-eating” amoeba *Naegleria fowleri* destroyed mouse embryo cells by cell nibbling as detected by immunofluorescence and electron microscopy. Later, Ralston et al. [17,23,24] demonstrated that *Entamoeba histolytica* (Eh), a diarrhea-causing protozoan parasite, possessed contact-dependent cell killing activity. This cell-contact killing activity was shown by biting off and ingest of host cell fragments termed “amoebic trogocytosis”. Furthermore, Somlata et al. [25] documented that AGC family kinase 1 was specifically involved in trogocytosis of living human cells but not participate in phagocytosis of dead cells by Eh. Recently, Bettadapur et al. [26] by using direct and high-throughput assay demonstrated that inhibition of human cell actin or amoeba surface Gal/GalNAc lectin could inhibit amoebic trogocytosis.

### 3.2. Immune Evasion Induced by Pathogenic Microbes via Trogocytosis

The acquisition of host membrane proteins by pathogenic microbes through trogocytosis may impact many host-pathogen interactions including immune evasion. Hereby, we will discuss the immune evasion mechanism in detail by pathogenic microbes in the following two subsections.

#### 3.2.1. Immune Evasion Induced by Eh via Trogocytosis

Many investigators demonstrated that the extracellular neutral cysteine proteinase secreted from Eh could degrade and prevent complement C3a, C5a and terminal complement complex attack [27,28,29]. Braga et al. [30] also showed that the galactose-specific adhesion of Eh could inhibit complement membrane attack complex effectively.

Begum et al. [31] explored that Eh could modulate and destroy host immune cells by inducing neutrophil apoptosis and stimulating respiratory burst and nitric oxide generation from macrophages. In addition, the adherence of Eh to host cells could mediate multiple cell cytotoxicity including promotion of cell death via phagocytosis, apoptosis, and trogocytosis those would play crucial roles in immune invasion [31]. Miller et al. [4] further confirmed that amoebic trogocytosis by acquisition and displaying human cell membrane proteins could facilitate escape from immune lysis by human serum.

#### 3.2.2. Intercellular Transfer of Intracellular Microbial Pathogens via Trogocytosis between Macrophages

Macrophages are the professional phagocytic and antigen-presenting immune cells capable of interacting with neighboring healthy cells via trogocytosis. Interestingly, a number of bacterial pathogens exhibit resistance to the anti-microbial activities and even grow within these cells. Steele et al. [32] found that the viable *Francisella tularensis* and *Salmonella enterica* bacteria could transfer from infected macrophages to uninfected macrophages together with cytosolic material through a transient mechanism resembling trogocytosis but without re-entering the extracellular space. While further exploring this unique immune evasion mechanism, the same authors discovered that *Francisella tularensis* bacteria acquired cellular components from infected macrophages and the embedded molecules within the double-membrane vesicles partially from the donor cell plasma membrane (trogocytosis) and their cytoplasmic components for escaping from immune attack [33]. In a similar way, B lymphocytes obtain avian flu H5N1 receptor, α2,3 sialic acid, from monocytes via trogocytosis for facilitating virus spread among immune cells despite lack of avian flu receptor on the B lymphocyte surface [34].

## 4. The Trogocytosis among Immune Cells for Information Exchange and Generation of Immune Plasticity in the Immune System

In multicellular organisms, trogocytosis appears firstly in mammalian immune cells where nibbling occurs at the immunological synapse [35]. After then, trogocytosis were successively discovered in T cells, B cells [36], NK cells [37], dendritic cells [38], macrophages [39], neutrophils [40], and basophils [41] those cells serve for modulating the immune responses [42].

Immunologically, trogocytosis represents the cell-cell communication and kiling of pathogenic microbes and mutated cells by the immune system by the following three aspects: (1) cell-cell communication and cell signaling; (2) killing of infectious agents, and (3) killing of endogenous cancer cells. In this subsection, we will only deal with the killing of viral pathogens via trogocytosis by the immune system in this subsection. The other two aspects will be discussed in the separate sections.

Rosenits et al. [43] explored the immune responses of T cells in vitro and in vivo to infection with lymphocytic choriomeningitis virus (LCM) and vaccinia virus. They used low frequencies of memory P14 T cells as an experimental cell model to show that T cells performed trogocytosis in vivo after contact with APC pulsed with GP33-peptide or expressing the antigen endogenously. The data showed that the trogocytosis-positive T cells expressed higher amount of activation biomarker and cytokines, suggesting a more activation compared to trogocytosis-negative T cells.

Similarly, Son et al. [44] also confirmed that effector and memory CD8^+^ T cells, but not naive CD8^+^ T cells, displayed more CD80 molecules on their surface obtained from antigen-presenting cells (APC) after acute LCM infection. Further analysis revealed that effector CD8^+^T cells expressed both intrinsic and extrinsic acquisition CD80 whereas memory CD8^+^T cell displayed only extrinsic acquisition. Interestingly, the extrinsic CD80 acquisition by CD8^+^ T cells was observed only in the lymphoid organs, but not in the periphery. These results may indicate that the trogocytosis of CD80 molecules is through the interactions between CD8^+^T cell and APC for the regulation of recall immune responses in memory CD8^+^ T cells. Tabiasco et al. [45] even documented that human natural killer (NK) cells could actively capture target cell membrane fragments in the site of immunological synapse controlled by Src kinase, ATP, Ca^2+^, PKC and actin cytoskeleton rearrangement.

**Figure 1:**
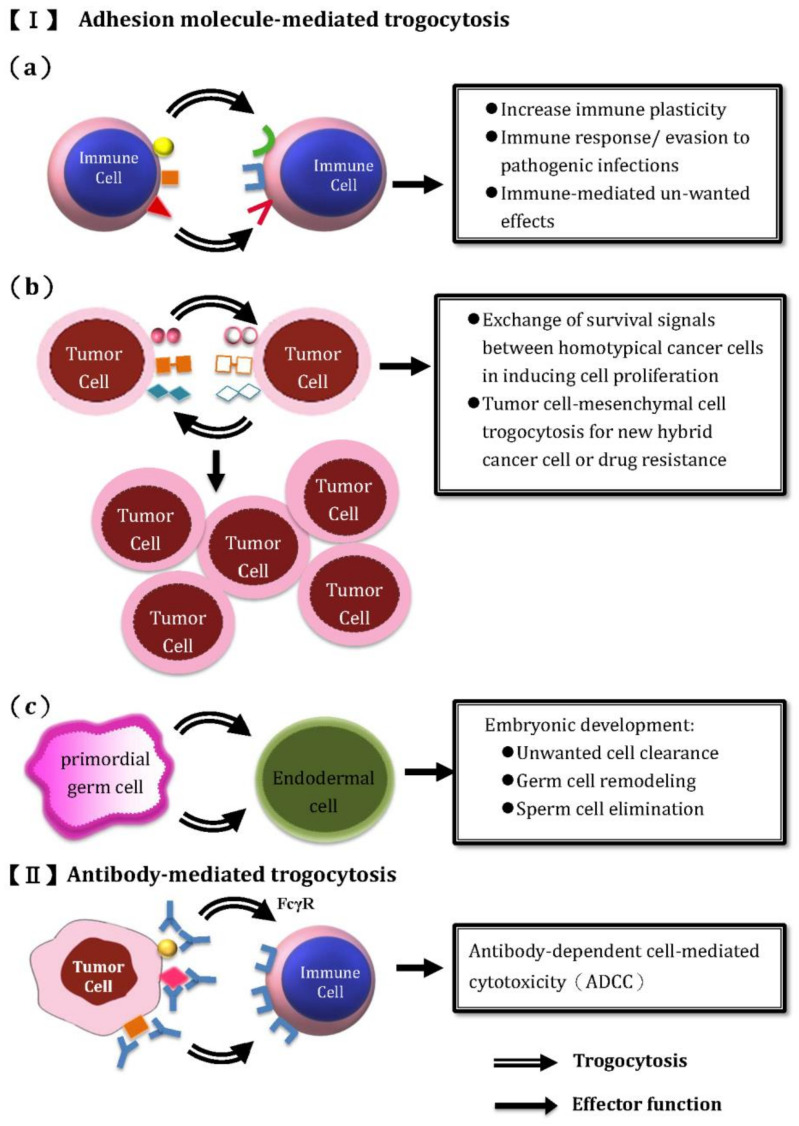
The classification of trogocytosis and its relevant effector functions by different immune and non-immune cells. (I) Adhesion molecule-mediated trogocytosis between: (**a**) immune cell- immune cell trogocytosis for modulating different immune responses and immune-mediated unwanted effects including allergic inflammation and cancer evasion. (**b**) Oncological trogocytosis for exchange of survival signals between homotypical tumor cells and tumor-mesenchymal cell trogocytosis for hybrid tumor cell formation or drug resistance. (**c**) Nibbling between primodial germ cell and endodermal cell for embryonic development via unwanted cell clearance. (II) Antibody-mediated trogocytosis amplifies the non-specific IgG FcγR-mediated binding and antibody- dependent cell-mediated cytotoxicity (ADCC).

In general, two mechanisms of trogocytosis have be identified: (1) adhesion molecule-mediated trogocytosis, and (2) IgG Fc receptor (FcgammaR)-mediated trogocytosis. The former occurs after adhesion of cell surface molecules on one cell with the specific ligands on other cells. The latter happens in the presence of antibody in bridging the antigens and FcgammaR on the acceptor cell. The intervention of the specific antibody in mediating trogocytosis via FcγR is characteristics. For detecting the trogocytosis between donor and acceptor cells, Daubeuf et al. [46,47,48], Wetzel et al. [49], and Cattapadhyay et al. [50] demonstrated that donor cells stained with suitable membrane fluorescent lipophilic probes and then detected by flow cytometry was a simple method for trogocytosis measurement. Then, the authors designed a trogocytosis analysis protocol (TRAP) for understanding the mechanisms and biological consequences of the acceptor cells (i.e., lymphocytes) after contact with APCs. The lipophilic fluorescent probes carrying either C_16_ or C_18_ saturated carbon chains in combination with intracellular staining were found suitable for TRAP assays [51]. The classification of trogocytosis and the relevant effector functions in embryonic development, immune system modifications, immune responses to infections, immune-mediated unwanted effects, and oncological trogocytosis are illustrated in Figure 1.

### 4.1. The Molecules on Immune Cells Involved in Trogocytosis

Many investigators have observed that membrane patches containing membrane-bound molecules were transferred from the cell surface of one immune cell to another one after immunological synapse formation in the murine system. Lorber et al. [52] demonstrated that the MHC class II I-A antigen on cloned alloreactive murine T lymphocytes are acquired passively. Hwang et al. [53] further demonstrated that T cell could use either T cell receptor or CD28 receptor to absorb and internalize the cell surface molecules from antigen-presenting cells. In human system, Ramming et al. [54] found that homotypic T-T interaction could induce T cell activation, proliferation and differentiation through interactions of activation-induced surface molecules (CD80, CD86, CD70 and MHC-class II) and their respective ligands constitutively expressed on the resting T cells. Then, membrane transfer of these activated ligands occurred among different combinations of the cell-cell interactions. Alegre et al. [55] further proved that human CD4^+^T, CD8^+^T and monocyte could acquire membrane patches and the intact protein molecules from different tumor cells by multiple simultaneous trogocytosis. Hudrisier et al. [56] found that a set of cell type specific determinants, including but not limited to antigen receptors, trigger trogocytosis. These determinants include TCR/CD3, co-receptors and costimulatory molecules on T cells involved in trogocytosis. On the other hand, the B cell receptors (BCR) and MHC antigens are potential triggers for B cell trogocytosis. It is also demonstrated that actin polymerization is more important in T rather than B cell trogocytosis. Aucher et al. [57] confirmed that actin polymerization and temperature were important for CD4^+^ and CD8^+^ T cells but no affection on B cells since B cell trogocytosis did not rely on active processes.

### 4.2. Intercellular Exchange of Membrane Patches Highlights the Next Level of Immune Plasticity

Immunobiologically, trogocytosis can play a key role in immune plasticity. Li et al. [58] demonstrated that trogocytosis between mononuclear cells (MNC) and PMN could transduce cell survival signals to suppress BAX, c-myc and caspase 8, and activating signals to induce p38 and P44/42-ARK-MAPK signals in both cells. Recently, Aucher et al. [57] reported that in vitro polarized CD4^+^ T cells were more efficiency in performing trogocytosis than Th1 or non-polarized CD4^+^T cells. Interestingly, these trogocytosis-positive polarized CD4^+^T cells generated in vivo displayed a Th2 phenotype [59,60]. These results indicate that trogocytosis-mediated signaling may play a crucial role in augmenting and shaping a Th2-dominant immune response. Furthermore, Li et al. [58] clearly showed that the spontaneous transfer of lactoferrins (LF) from PMN to skew autologous CD4^+^ T toward Th2 differentiation after cell-cell contact.

However, Riond et al. [61] by using a model of viral antigen LCMVgp33-41 recognition in transgenic P14 mice showed that CD8^+^ T cell underlying trogocytosis in vivo after interactions with target cells and dendritic cell could express activation marker CD69. The authors suggested that trogocytosis may become a novel in vivo biomarker of the recent interaction between CD8^+^T cells and its cellular targets.

Recently, Nakashima et al. [62] reported that CD30 and CD30L trogocytosis between mCherry-CD30L/CHO cells and CD30-HeLa cells resulted in the generation of signalosomes, intracellular signaling, lysosomal degradation and a subsequent refractory phase of malignant cells. This result may indicate that the signaling pathway can be initiated by ligand-receptor trogocytosis in both normal and malignant cells.

## 5. Trogocytosis between Basophils and Dendritic Cells in Supporting Th2-Mediated Allergic and Inflammatory Reactions

Basophils are the least abundant granulocytic population accounting for less than 1% of leukocytes in the blood. In addition to playing a crucial role in allergy, Sokol et al. [63] reported that basophils were important for the development of Th2 cells by acting as an IL-4 provider. Yoshimoto et al. [64] further demonstrated that administration of antigen-pulsed basophils, but not antigen-pulsed DC or mast cells, induced Th2 cell differentiation in vivo. These data may suggest that basophils act as Th2-inducing APCs in the development of allergic reaction. In vitro experiments also demonstrated that basophils strongly produced IL-4 and IL-13 in response to IL-3, IL-18 or IL-33 contributing to the development of Th2 immune responses [65,66]. Although basophils express little or no MHC-class II proteins in the cell surface, they can acquire peptide-MHC-II complexes from dendritic cells via trogocytosis. In association with IL-4 production, basophils can affect naive CD4 T cells skewing to Th2 cell differentiation [67]. Karasuyama et al. [68] also demonstrated that activated basophils could release serine proteases, mouse mast cell protease 8 (mMCP-8) and protease 11 (mMCP-11) to elicit microvascular hyperpermeability and leukocyte infiltration in the affected tissues leading to allergic inflammation in the affected tissues.

**Figure 2:**
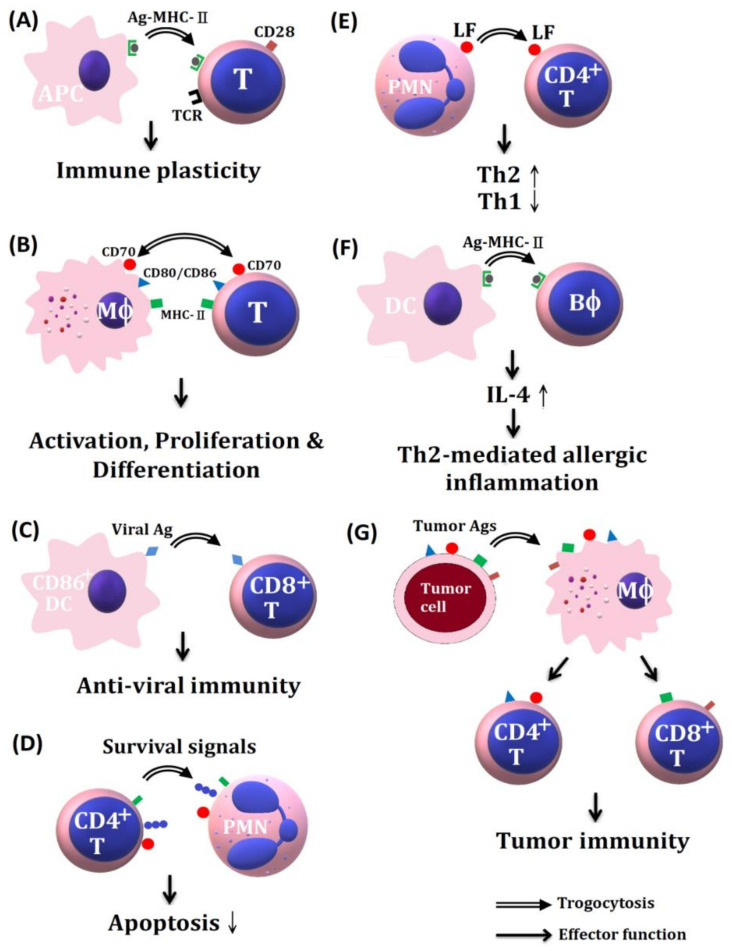
The trogocytosis of various membrane molecules between different innate and adaptive immune cells or tumor cells in mediating a variety of effector function. (**A**) Trogocytosis of antigen-MHC II complex from APC to TCR on T cells in inducing immune cell plasticity. (**B**) The transfer of MHC II and CD80/86 molecules from macrophages to T cells sustains T cell activation, proliferation, and differentiation. (**C**) The transfer of viral antigens from CD80+ DC to CD8+T cells can induce anti-viral immunity. (**D**) CD4+T cells transfer survival signals to PMN in suppressing PMN apoptosis. (**E**) The transfer of lactoferrins from PMN to CD4+T cells can skew CD4+ T cells toward Th2 immune responses. (**F**) The transfer of antigen-MHC II complex to basophils can enhance IL-4 production inducing Th2-mediated allergic reaction. (**G**) The transfer of tumor-specific antigen from tumor cells to macrophages and then distributes to both CD4+T and CD8+ T to mediate tumor cytotoxicity. Ag-MHC: antigen conjugated-major histocompatibility complex, APC: antigen-presenting cell, DC: dendrite cell, PMN: polymorphonuclear neutrophil, LF: lactoferrin, Bϕ: basophil, Mϕ: macrophage.

A scheme illustrating the effects of trogocytosis-related molecules among different immune-related cells in immune responses and tumor immunology is shown in Figure 2.

## 6. The Effects of Trogocytosis on the Allogeneic Transplantation in Maintaining Allograft Survive

Ohlen et al. [69] demonstrated that human donor cells transplanted into SCID mice were under constant immune surveillance by NK cells and macrophages, but adapted to immune evade detection. Yamanaka et al. [70] further demonstrated that human donor cells were protected from NK cell-mediated killing by the recipient mice in that these donor cells obtained mouse (host) MHC class I molecules for triggering the recipient NK cell inhibitory receptor. Accordingly, the transfer of host MHC class I protein via trogocytosis to donor cells can protect donor cells from NK and macrophage-mediated rejection in hematopoietic stem cell transplantation [71,72]. Therefore, the in utero hematopoietic cellular transplantation (IUHCT) might become a promise therapeutic strategy for the treatment of congenital disorders. On the other hand, Durkin et al. [73] reported that the exposure to donor ligands led host immune system recognize donor alloantigens as self and drive NK cell selection. The same group further examined donor-to-host MHC transfer as an intrinsic mechanism in regulating the development and maintenance of NK cell tolerance in prenatal chimera [74]. These authors clearly demonstrated that the phenotypically tolerant host NK cells expressed low levels of transferred donor MHC complexes during fetal development. This chimerism persisted tolerable to the later mature cytotoxic lymphocytes in pregnancy. However, the precise molecular mechanism of how these cis-regulation of transferred donor MHC molecule affecting the maintenance of tolerant NK cells in prenatal chimera needs further investigation.

## 7. Trogocytosis among Immune-Related Cells and Tumor Cells in Mediating Immune Surveillance and Immune Evasion

Immune responses against foreign pathogens or endogenous mutated cells require fine immune regulation. Accordingly, exchange of membrane molecules/antigens among innate and adaptive immune cells can fine-tune the optimal immune reactions to protect body from these noxious invaders [75,76,77,78,79]. On the other hand, the endogenous mutated tumor cells can exhibit their defense strategies to escape from immune surveillance or end in immune evasion. The cellular defensing mechanisms of both sides can be achieved by trogocytosis among immune-related cells and tumor cells or modulating immune-tumor interactions. We will discuss in detail on the roles of trogocytosis between immune-related cells and tumor cells for tumor immunology in the following subsections.

### 7.1. Capture of Tumor Cell Membranes by CD8^+^ Cytotoxic T Lymphocytes, NK Cells and Chimeric Antigen Receptor T Cells (CART) for Tumor Surveillance

It is well-documented that the success of adoptive immune cell transfer in the treatment of metastatic cancer in human is dependent on the selection of highly active tumor specific cytotoxic T lymphocytes (CTLs). These tumor-specific CTLs have been shown to acquire MHC antigens from antigen-presenting cells [41] at immune synapse [80]. Machlenkin et al. [81] demonstrated that in a human melanoma in vivo model adoptive transfer of membrane-trogocytosed peptide-specific T cells, but not non- capturing CD8^+^T cells, could potently inhibit tumor progression. Uzana et al. [82] studied the functional diversity within tumor-specific T cell clones with identical TCR specificity in melanoma. The authors found that the high-active T cell clones displayed prolonged phosphorylation of ribosomal protein S6 (an integrator of MAPK and AKT activation) whereas the low-active T cell clones generated shorter and weaker phosphorylation. They concluded that trogocytosis is a gateway to define functional diversity in melanoma-specific CD8^+^ cytotoxic T cell clones. Following this line, the same group further demonstrated that CD8^+^T-APC interactions could directly kill T cells with the same or different TCR specificity after trogocytosis. This result may indicate that trogocytosis enables cross-reactivity among CD8^+^T cells with dual roles as effectors and APCs to amplify or restrict anti-tumor immune response [83].

Natural killer cells possess the ability to recognize and kill tumor cells, rendering these cells ideal candidates for tumor immunotherapy [84]. Williams et al. [85] reported that transfer of small membrane patches in the immune synapse to the partner cell occurred after NK-target cell interaction. Therefore, the molecules on the target cell can appear on the surface of NK cells. Marcenaro et al. [86] found that the transfer of chemokine receptor CCR7 from donor cells to NK cells via trogocytosis could active NK cell migration and enhanced lymph node homing. Furthermore, Domaica et al. [87] reported that NKG2D and NKp46 ligands captured by T cells through trogocytosis could promote NK cell cytotoxic to tumor cells. Cho et al. [88] firstly demonstrated increased NK cell cytotoxicity after the acquisition of chimeric antigen receptors (CARs) on CAR^+^T lymphocytes (CART) through trogocytosis. CARs are the synthetic antigen receptors that can reprogram T cell specificity and function. The persistent CART cells derived from patients have been demonstrated remarkable efficacy against a range of B-cell malignant lymphoma [89]. In addition, Brudno et al. [90] recently demonstrated that genetically modified T cells expressing an anti-B cell maturation antigen with chimeric antigen receptor elicited remission in poor-prognostic relapsing multiple myeloma. However, relapse occurred in a large fraction of patients with multiple myeloma, some of them were antigen-negative and others were antigen-low [91]. For exploring the molecular mechanism of high relapsing rate in CART therapy, Hamieh et al. [92] disclosed that CART cells provoked reversible antigen loss through trogocytosis. Thereby, decreasing target antigen density on tumor cells and abating T cell killing activity attributed to decreased T cell killing capacity and enhanced T cell exhaustion. This evidence may suggest trogocytosis by CART plays a double-edged sword in cancer immunotherapy.

**Figure 3:**
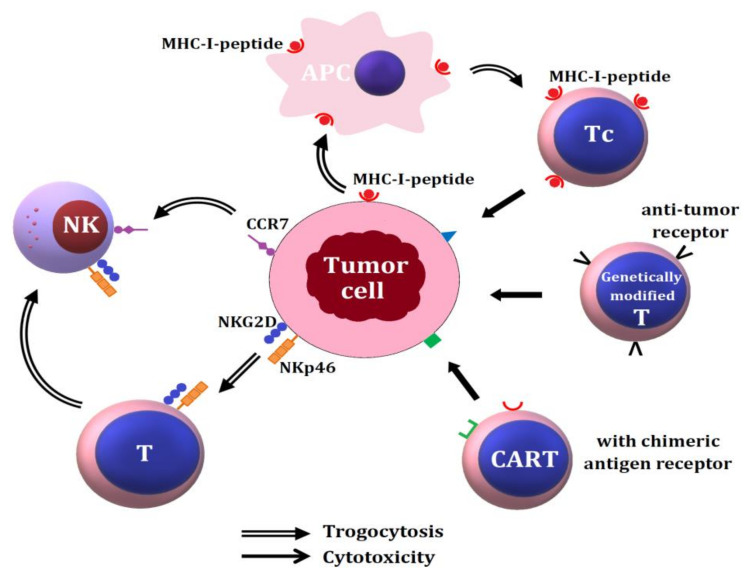
The roles of trogocytosis among tumor cells and different immune-related cells in tumor immunity. The MHC-I-peptide complex expressed on tumor cells is trogocytosed by APC and then transferred to cytotoxic T cells (Tc) for tumor surveillance and cytotoxicity. The surface-expressed NKG2D and NKp46 molecules on tumor cell are trogocytosed by T lymphocyte and then are transferred to NK cells for tumor killing. The chemotactic receptor CCR7 on tumor cells can be trogocytozed by NK cells for direct cytotoxicity. In addition, the genetically modified and chimeric antigen receptor (CAR)-T lymphocytes (CART) can directly kill tumor cells by expressing the specific anti-tumor receptors for binding with the tumor antigens.

A scheme demonstrating increased tumoricide activity after capture of tumor cell membrane antigens by immune cells via trogocytosis is shown in Figure 3.

### 7.2. Antibody-Opsonized Tumor Cell Killing by Innate Immune Cells and Augmented by Trogocytosis and Other Immune-Mediated Mechanisms

In addition to immunological checkpoints inhibitors to restore the T cell anti-cancer immunity [93] and the above mentioned CART cells for cancer immunotherapy, two strategies have been designed recently to reactivate innate immunity against tumors by: (1) activating macrophages or polymorphonuclear neutrophils with anti-CD47 antibody, and (2) use of anti-tumor antibody to induce antibody-dependent cell-mediated cytotoxicity (ADCC) by innate immune cells. These two mechanisms can be augmented via trogocytosis. However, the use of anti-tumor antibody may also shave the tumor antigens and conversely enhance tumor chemo-resistance.

#### 7.2.1. Reactivation of Anti-Cancer Immunity of Macrophages by Using Anti-CD47 Antibody and Other Anti-Cancer Antibodies with Augmentation by Trogocytosis

Brown et al. [94] discovered that integrin-associated protein CD47 was a widely expressed trans-membrane protein in phagocytes and dendritic cells serving as the ligand for signal regulatory protein SIRPα. The activation of CD47 initiates a signaling cascade to inhibit phagocytosis [95]. Clinically, Majeti et al. [96] found that CD47 is an adverse prognostic factor and could become an effective therapeutic antibody target for human acute myeloid leukemia stem cells. Weiskopf et al. [97] demonstrated that anti-CD47 blocking antibody could activate macrophage-mediated destruction of human small-cell lung cancer. However, Kennedy et al. [98] noted that the infusion of certain anti-tumor cell antibodies including rituximab, trastuzumab, cetuximab or mAbT101 could induce target epitopes loss. This phenomenon is called “antigenic modulation” and may compromise monoclonal antibody-based anti-cancer therapies. Alternatively, Beum et al. [99] explored that antigenic modulation could promote trogocytosis by monocytes/macrophages after antibodies binding to cancer cells. Boross et al. [100] further demonstrated that both activating and inhibitory IgG-Fcγ receptors (IgG-FcR) would mediate rituximab-induced CD20 trogocytosis by macrophages in mice experiments. Theoretically, antigenic modulation during anti-CD20 antibody therapy can probably impair the efficacy of immunotherapy and results in “pro-tumorigenic” state by escape from antibody attack. However, Velmurugan et al. [101] conversely demonstrated that the persistent macrophage-mediated trogocytosis via IgG-FcR led to kill more of the antibody-opsonized tumor cells despite antigen depletion.

#### 7.2.2. Antibody-Opsonized Cancer Cell Killing by ADCC of Innate Immune Cells and Augmented by Trogocytosis

Neutrophils comprise around 60% of all white blood cells in the circulation and are widely recognized to play a modulatory role in cancer immunity dependent on the context and cancer stage [102,103]. Anti-tumor effect of PMN is found in the early stages of tumorigenesis [104] whereas a pro-tumoral role was reported at late stages of cancer [105].

Horner et al. [106] demonstrated that PMN and target cell contact activated mutual membrane lipids exchange and enhanced cytotoxicity in the presence of tumor-targeting antibodies. This tumoricidal activity of PMN is related to ADCC. Furthermore, Matlung et al. [107] found that both anti-tumor antibody and IgG-FcγR-dependent cancer killing activity by PMN could be augmented by trogocytosis and CD47-SIRPα checkpoint inhibitors. Besides, Treffers et al. [108] even disclosed that IgA antibodies were superior to IgG antibodies in killing cancer cells by PMN in combination with CD47-SIRPα checkpoint inhibitor. A comprehensive review of neutrophils in fighting against cancers has been reported by Usyannovska Avtenyuk et al. [109].

**Figure 4:**
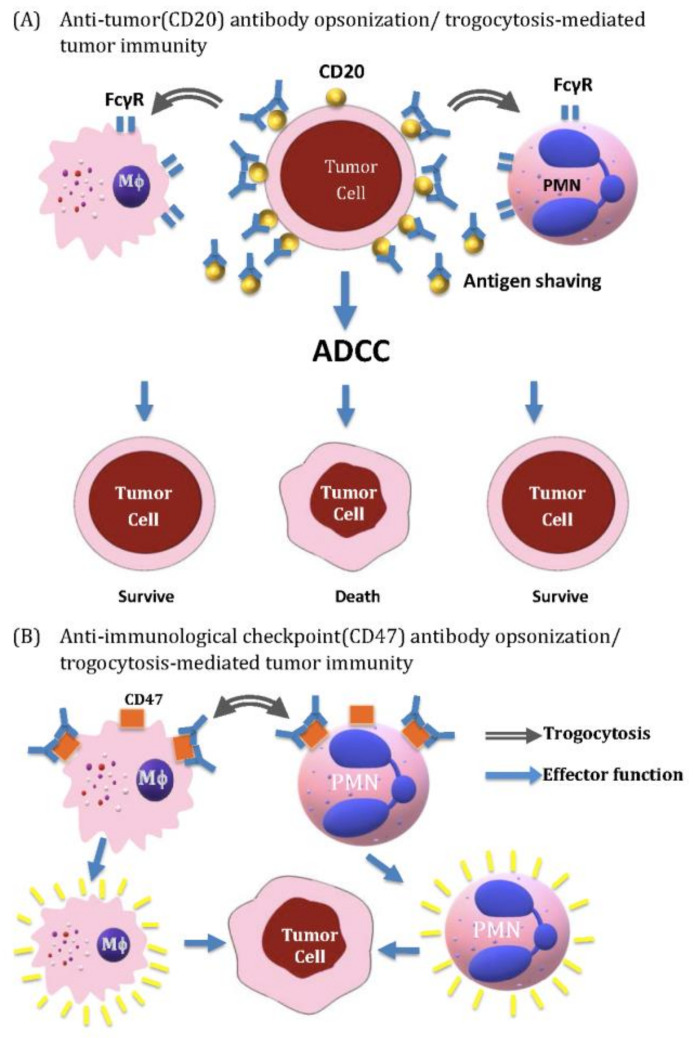
Anti-tumor antibody (anti-CD20)-opsonized tumor cells (**A**) and anti-immunological checkpoint antibody (anti-CD47)-opsonized innate immune cells (**B**) in mediating tumor immunology. (**A**) Anti-CD20 antibody can mediate tumor-killing activity by direct antibody-dependent cell-mediated cytotoxicity (ADCC). In addition, the anti-CD20 attached-CD20 antigens on tumor cells can be transferred to macrophages and PMNs via trogocytosis for further sensitization in both innate immune cells to augment their tumor-killing activity. However, the shaving of ani-CD20 attached- CD20 antigens from tumor cells decreases the tumor antigen density and may escape from further anti-CD20 antibody attack. (**B**) anti-CD47 blocking antibody can mediate tumor-killing directly by activating both macrophages and PMNs and be further augmented by mutual trogocytosis.

A scheme depicting tumor-killing activity by innate immune cells via ADCC and augmented by antibody-opsonized trogocytosis, but suppressed by antibody-mediated antigen shaving is shown in Figure 4.

#### 7.2.3. Immune Evasion via Trogocytosis of Immunosuppressive Molecules among Tumor Cells, Immune-Related Cells, and Mesenchymal Stroma/Stem Cells (MSC)

Poupot et al. [110] observed that some leukemic cell lines, including Daudi Burkitt lymphoma cells, exhibited continuously constitutive synaptic patches transfer between homotypic cells. This intercellular transfer required cell-cell contact through immunological synapse and involved constitutive protein kinase C and MAPK/ERK activities. Then, the strong B cell receptor activation and autoreactivity ensued. Later, Le Maoult et al. [111] investigated the trogocytosis among hematological cell lines and the freshly isolated hematological tumor cells. The authors demonstrated that these cell lines as well as the isolated tumor cells possessed a trogocytotic capacity to capture membranes containing a immune-inhibitory molecule HLA-G from allogeneic sources. This result supports the fact that trogocytotic capacity of liquid tumor cells could escape from immune attack through transfer of the membrane-bound immune inhibitory molecule HLA-G to immune-related cells. HLA-G is a non-classical HLA class I antigen widely expressed in various malignant tissues with immune suppressive functions. We will discuss in detail on the role of HLA-G and the oncologic trogocytosis among tumor cells, immune-related cells, and mesenchymal stroma/stem cells in the immune evasion in the following subsections.

##### 7.2.3.1. Trogocytosis of HLA-G from Tumor Cells to NK Cells, Macrophages and T Cells

NK cells serve as a first-line defense mechanism against tumors in lysing MHC class-I negative targets [112,113]. However, HLA-G expressing tumors can conversely inhibit NK cell cytotoxicity by interfering immunoglobulin-like transcript 2 (ILT-2) and killer immunoglobulin-like receptor (KIR2DL4, CD158d) [114]. Caumartin et al. [115] showed that activated NK cells captured HLA-G1 molecules from tumor cells via trogocytosis in minutes. The acquisition renders NK cells temporarily stop proliferation and cytotoxicity, and induction of suppressor NK cells to protect NK-sensitive tumors from cytolysis. In addition, The HLA-G molecules on tumor cells can be transferred to monocytes and T cells via trogocytosis. However, the expression on monocytes is much shorter than that of T lymphocytes. Accordingly, the suppressive function of HLA-G (+) on monocytes is rapidly lost compared to T cells [116].

Many authors [117,118,119,120] demonstrated that both HLA-G and B7 molecule CD86 are the markers of poor prognosis when present on multiple myeloma cells because the two molecules could be trogocytosed by both CD4+T and CD8+T cells to inhibit cell proliferation, similar to suppression by natural regulator T cells. After transfer of HLA-G from cancer cells to immune-related cells, the molecule would interact with inhibitory receptors ILT2 and ILT4 on immune cells to transduce complex suppression signals leading to immune evasion [121].

##### 7.2.3.2. Trogocytosis of PD-L1/L2 Molecules among Tumor Cells, APC and CD8+ T Cells in Facilitating Immune Evasion to Tumor Cells

The programmed cell death 1 (PD-1) receptor on T cells is capable of specifically interacting with programmed cell death ligand 1 (PD-L1) on APC to transduce the inhibitory signals to suppress T cell responses [122]. The activation of PD-L1 on APC can activate APCs to produce TGF-beta. This suppressive-type cytokine then facilitate differentiation and maintenance of the inducible regulatory T cells (iTreg) in mice by interfering the mTOR-Akt signaling pathway in the effector T cells [123]. Furthermore, the iTreg stimulates PD-L1 expression on DCs to become suppressor DCs [124]. Hino et al. [125] found that PD-L1 was up-regulated on a broad variety of human cancer cells, indicating PD-1/PD-L1 signaling pathway may be involved in the immune evasion of tumor cells. Gary et al. [126] demonstrated that human antigen-specific CD8+T cells acquired PD-L1 molecule from mature dendritic cells and tumor cells via trogocytosis in an antigen-specific manner. Of importance, these CD8+T cell were then able to induce neighboring PD-1 expressing CD8+T cell apoptosis. These observations strongly indicate that the transfer of functionally active co-inhibitory molecules PD-L1 from APC to CD8+T cell would play a regulatory role in immune responses. Following this line, Kawashima et al. [127] co-cultured Hodgkin Reed-Sternberg (HRS) cells with monocytes and found PD-L1/L2 expression were elevated rapidly in monocytes within 1 h after trogocytosis. The rapid elevation of PD-L1/L2 in monocytes can potently suppress T cell response elicited by MHC antigen presentation from tumor cells to evade anti-tumor immunity.

**Figure 5:**
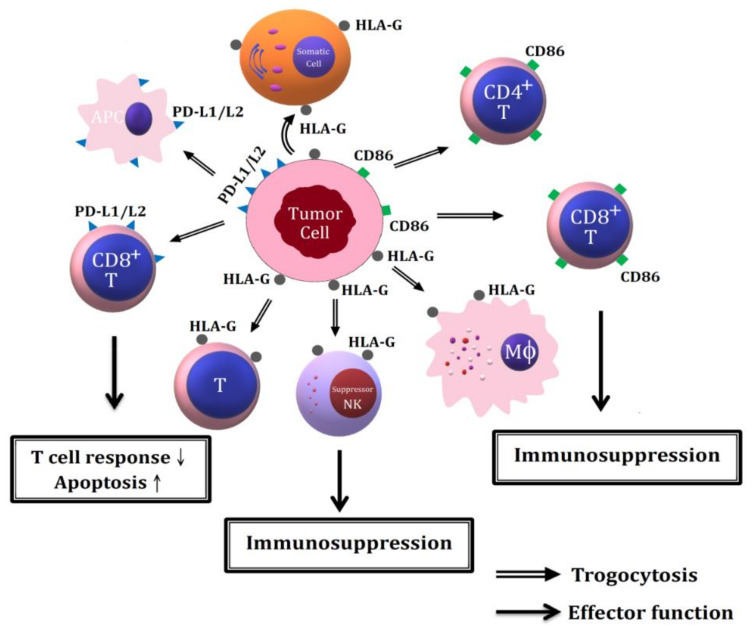
The molecular basis of tumor evasion from immune system via trogocytosis of immune suppressive molecules including HLA-G, PD-L1/L2 and CD86 on tumor cells to immune-related cells. The trogocytozed PD-L1/L2 from tumor cells to APC and CD8^+^T cells suppress T cell response but enhance T cell apoptosis. The trogocytosis of HLA-G from tumor cells to macrophages, T cells and NK cells leads to immunosuppression and tumor cells immune evasion. The trogocytosis of CD86 molecules from tumor cells to CD4^+^T and CD8^+^T leads to immunosuppression and tumor cells immune evasion. PD-L1/L2: programmed cell death ligand1/ligand 2.

The effects of trogocytosis of different membranous immunosuppressive molecules from tumor cells to different immune-related cells in mediating immune invasion is illustrated in Figure 5.

##### 7.2.3.3. Oncologic Trogocytosis between Tumor Cells and Microenvironmental Mesenchymal Stroma/Stem Cells in Increasing Tumor Heterogeneity

A growing amount of studies are undergoing to disclose the oncologic trogocytosis between epithelial carcinoma cells and microenvironmental MSC. Rafii et al. [128] found that epithelial ovarian cancer cells captured patches of the “hospicells” (original type of stromal cells) through oncologic trogocytosis. The acquisition of these functional P-glycoproteins (P-gp) from hospicells renders cancer cell developing chemoresistance. Yang et al. [129] explored that the exchange between tumor cells and MSC significantly increased epithelial adhesion molecules and gene expression for cytokeratins and epithelial-like differentiation factor in MSC. Conversely, a variety of transcriptional regulatory genes were down-modulated in tumor cells after co-culture with MSC. Ultimately, the cross-talk between MSC with cancer cells via trogocytosis promotes cell fusion by forming new cancer hybrid cells with heterogeneity [130,131,132].

## 8. Abnormal Trogocytosis in Systemic Autoimmune and Immune-Mediated Inflammatory Diseases

It is conceivable that trogocytosis among immune cells exerted an important influence on the course of immune responses by either stimulatory or suppressive effect. He et al. [133] found that CD4+T cells captured surface membrane MHC-II and the neighboring immunological synapse molecules including costimulatory CD40, CD54, CD80, OX40L and 41BBL on APC. These trogocytosed molecules retained on the T cell surface in association with TCR for activating ZAP-70, phosphorylated tyrosine and ERK1/2 to sustain TCR signaling [134]. However, when the number of the activated T cell increases, the T-T cell interactions would lead to cell apoptosis and induction of anergy, tolerance, and production of regulatory T cell [135,136]. Arbitrarily, there are two mechanisms involved in mediating cell-cell trogocytosis: (1) adhesion molecule-mediated trogocytosis through cell-cell synapse formation [137], and (2) IgG-FcR-mediated trogocytosis [56,138]. A major difference of FcγR-mediated trogocytosis from adhesion molecule-mediated trogocytosis is the intervention of antibody [138]. Besides, Haastert et al. [139] found that T cells at the site of autoimmune inflammation exhibited increased potential for trogocytosis. As discussed in Section 7.2.3.1. decrease in expression or aberrant trogocytosis of HLA-G in immune cells can induce autoimmune reaction.

### 8.1. Decreased Expr#Ession of HLA-G Mole#Cule on # in the Patients with Systemic Lupus Erythematosus (SLE)

Biologically, HLA-G proteins contain 7 isoforms from HLA-G1 to G4 are membrane-bound while HLA-G5 to G7 exist as soluble proteins generated by alternative splicing [140,141]. However, the HLA-Gs expression is restricted in certain tissues such as placental cytotrophoblasts, thymus, eyes [142,143,144], and human peripheral blood mononuclear cells [145]. These molecules exhibit a regulatory role through its interaction with ILT2 and ILT4, and KIR2DL4 [146,147]. LeMaoult et al. [148] showed that the expression of HLA-G on APC could suppress CD4+ lymphocytes proliferation stimulated by allogeneic cells. Monsivais-Urenda et al. [149] found that monocytes and mature CD83^+^DCs expressed a diminished HLA-G in patients with SLE compared to healthy controls. Besides, the authors found that IL-10-activated SLE monocytes also expressed diminished amount of HLA-G. Functional analysis of IFN-γ activated SLE-monocytes exhibited a decreased ability to suppress autologous lymphocyte proliferation. Furthermore, SLE-lymphocytes displayed a lower HLA-G trogocytosis with autologous monocytes. The authors concluded that the expression and function of HLA-G on immune cells are decreased in patients with SLE. However, Rosado et al. [150] and Negrini et al. [151] reported that the percentage of HLA-G expressing cells on PBMC was significantly increased in SLE patients. These authors argued that the up-regulated HLA-G membrane expression on PBMC might reflex a compensatory down-regulation on hyperactive immune state in patients with SLE. Nevertheless, the real cause for the discrepancy among these studies remains elucidation. For controlling the hyperactive immune state of SLE, treatment with bi-specific hexavalent anti-B cell antibody comprising epratuzumab and veltuzumab (humanized anti-CD20 monoclonal antibody) for controlling B cell hyperreactivity can enhance FcγR-mediated trogocytosis to shave the B cell surface antigens including CD19, CD20, CD21, CD22, CD79b, CD44, CD62L and B7 integrin as shown in Figure 5. This new therapeutic strategy may suppress the B cell antigen-presenting capacity in autoimmune responses of patients with SLE [152].

### 8.2. Aberrant Trogocytosis in Other Autoimmune and Immune-Mediated Inflammatory Diseases

Skeletal muscle is the largest tissue of the body and becomes the target of many immune reactions such as autoimmune myopathies [153]. Arahata et al. [154] discovered intimate interaction of T cells with muscle fibers at sites of inflammation in muscle biopsy specimens in patients with autoimmune myopathy. Waschbisch et al. [155] found that human myoblasts donated membrane fragments to activate T cell proliferation. The membrane transfer between myoblasts and T cells is sensitive to actin polymerization inhibitor and is augmented by PKC activation. Therefore, the trogocytotic mechanism may become a potential target for modulating muscular immune responses in the patients with inflammatory muscular diseases.

Clark et al. [156] reported that pure IgA autoantibody-coated RBCs were destroyed by monocytic phagocytosis rather than complement-mediated hemolysis in patients with autoantibody-induced hemolytic anemia (AIHA). Chadebech et al. [157] discovered that pure IgA autoantibody-opsonized RBCs could transfer RBC membrane to monocytes contributing to immune-mediated RBC destruction. Furthermore, this destruction could not be inhibited by blocking FcαR1. These evidences may suggest that trapping and subsequent sequestration of pure IgA antibody-agglutinated RBC in the spleen is augmented principally by trogocytosis.

Obesity is characterized by the chronic low-grade activation of the innate immune system with release of adipocytokines such as Il-6, TNF-α and MCP-1. These pro-inflammatory cytokines can further stimulate macrophage infiltration into adipose tissues [158]. By using time-lapse microscopy, flow and laser-scanning cytometry, Sarvari et al. [159] observed that macrophages could trogocytose bites of adipocytes at the sites of adipocyte-macrophage interaction. These trogocytosed macrophages activated NF-κB translocation, and then released IL-6 and MCP-1, but not other proinflammatory cytokines such as TNF-α and IL-8. The authors concluded that the trogocytosis-dependent IL-6 secretion had significant regulatory functions in the inflamed adipose tissue. The adipocyte-macrophage interactions in obesity may also increase macrophage type 1 (M1)/type 2 (M2) cell ratio and CD11b^+^F4/80^+^CD11c^+^ -triple positive adipose tissue macrophage number [160]. Recently, Lu et al. [161] demonstrated that MAP kinase phosphatase-5 (MKP-5) could switch macrophages from M1 to M2 phenotype and became an inflammatory inhibitor in obese-derived adipose tissue inflammation through P38, JNK and ERK-MAPK signaling pathways.

The abnormal trogocytosis-mediated adverse effects in different autoimmune and immune-mediated inflammatory diseases are summarized in Table 1.

## 9. Conclusions and Perspectives

Cell membrane molecule transfer is one of the cell-cell interactions for information exchange among homotypic or allogeneic cells after cell contact. Two major mechanisms can be identified, including adhesion molecule-mediated and IgG-FcR-mediated trogocytosis. The transferred membrane molecules on different cells may be involved in cell survival, anti-microbial activity, immune plasticity, cell activation/proliferation/ differentiation, anti-tumor immunity/tumor evasion, and embryonic development. However, aberrant trogocytosis in the immune system may lead to autoimmune, inflammatory and allergic diseases. Recently, applications of antibodies in the treatment of autoimmunity or tumor immunotherapy have an opportunity to shave the antigens (antigen modulation) via trogocytosis in enhancing drug resistance. In addition, the oncological trogocytosis between tumor cells and microenvironmental mesenchymal stroma/stem cells can promote cell-cell fusion and subsequently facilitates cancer hybrid cell formation with plasticity. New therapeutic strategy should be considered to overcome these problems in the future cancer immunotherapy. The following enigmas still remain in the field of trogocytosis research:

(1) The real molecular mechanism of cell-cell membrane transfer and the repulsive force after cell-cell contact.
(2) The real molecular mechanism of homotypic tumor-tumor and tumor-MSC trogocytosis in hybrid cancer cell formation and for novel cancer therapy in future.
(3) The appropriate use of cytoskeletal protein blockade to suppress trogocytosis of immunomodulatory molecules (HLA-G, PD-L1/L2 and CD37) between tumor and immune cells.
(4) The selective modulation of stimulatory or inhibitory IgG-Fc receptor expression in adjusting cell-cell membrane transfer remains elucidation.
(5) The best strategy of antibody-opsonized tumor cell killing by immune-related cells remain evaluation.

Although anti-tumor and anti-immunological checkpoint antibodies can effectively mediate tumor killing via ADCC or immunopotentiation by immune –related cell as discussed in Section 7, antigen shaving and FcgammaR-expressing cell exhaustion would diminish the efficacy. Velmurugan et al. [162] explored that antibody engineering could increase FcγRs affinity and enhanced tumoricidal activity. In addition, Li et al. [163] demonstrated that engineered antibodies interacted with both IgG and IgA Fc receptors on monocytes/macrophages would improve tumor immunotherapy. Recently, Li et al. [164] by employing peptide-MHC (pMHC) multimer technology to probe TCR ligands on tumor cells could identify new target antigens for tumor immunotherapy.

(6) CART immunotherapy may also face several challenges including therapeutic potency, impaired trafficking to solid tumor, local immunosuppression within tumor microenvironment, and toxicity associated with CART cells. Although Petty et al. [165] proposed to use CAR-NK or CAR-NKT cell instead of CAR-T cells. Combination therapy of CAR cells and anti-tumor antibody may potentially improve the efficacy for CAR cells trafficking into solid tumors.

## Figures and Tables

**Table 1:** Abnormal trogocytosis and the aberrant transferred molecules in patients with autoimmune, inflammatory diseases and obesity.

Disease Entity	Abnormal Trogocytosis-Mediated Adverse Effects
Systemic lupus erythematosus	-Decreased HLA-G molecule expression on monocytes and CD83^+^ dendritic cell [149] -Diminished lymphocytes trogocytosis of HLA-G molecules from autologous monocytes [149] -Increased % of HLA-G expressing PBMC reflects a compensatory down-regulated hyperactive immune status [150,151]
Autoimmune myopathy	-Increased trogocytosis between myoblasts and T lymphocytes [155]
IgA autoantibody –mediated hemolytic anemia	-Increased RBC membrane trogocytosis to monocytes and sequestration in spleen [157]
Obesity	-Increased adipocyte to macrophage trogocytosis in enhancing IL-6 and MCP-1 production by macrophages [158,159]

## Data Availability

Not applicable.

## Abbreviations

ADCC	antibody-dependent cell-mediated cytotoxicity
AIHA	antibody-induced hemolytic anemia
APC	Adenosine diphosphate ribose
Bϕ	basophil
CART	chimeric antigen receptor T lymphocyte
CCR	C-C chemokine receptor
CD4^+^ T	helper T lymphocyte
CD8^+^T	cytotoxic T lymphocyte
CTL	cytotoxic T lymphocyte
DC	dendritic cell
Eh	*Entamoeba histolytica*
Gal/GalNAc	galactose/N-acetyl-galactose
HLA-G	human leukocyte antigen-G
iTreg	inducible regulatory T cell
IL	interleukin
ILT	immunoglobulin-like transcript
IUHCT	in utero hematopoietic cellular transplantation
KIR	killer immunoglobulin-like receptor
MCP	macrophage chemotactic protein
Mϕ	macrophage
MHC-I	major histocompatibility complex class I antigen
MHC-II	major histocompatibility complex class II antigen
MSC	mesenchymal stroma/stem cell
NK	natural killer cell
PGC	primordial germ cell
PD-1	programmed cell death-1
PD-L1/L2	programmed cell death ligand 1/2
SCID	severe combined immunodeficiency
SLE	systemic lupus erythematosus
Tc	cytotoxic T lymphocyte
Th1	helper T cell type1
Th2	helper T cell type2
